# Dermaseptins, Multifunctional Antimicrobial Peptides: A Review of Their Pharmacology, Effectivity, Mechanism of Action, and Possible Future Directions

**DOI:** 10.3389/fphar.2019.01421

**Published:** 2019-11-26

**Authors:** Emiel Jacob Henri Bartels, Douwe Dekker, Mohamed Amiche

**Affiliations:** ^1^University Medical Center, University Utrecht, Utrecht, Netherlands; ^2^Dutch Poisons Information Center, University Medical Center Utrecht, Utrecht, Netherlands; ^3^Mondor Institute of Biomedical Research, INSERM U955 Team 7, School of Medicine, University Paris Est Créteil, Créteil, France

**Keywords:** dermaseptin, *Phyllomedusa bicolor*, peptide, amphibian, infectious, antimicrobial, tumor

## Abstract

Dermaseptins are a group of α-helical shaped polycationic peptides isolated from the Hylid frogs, with antimicrobial effects against bacteria, parasites, protozoa, viruses *in vitro*. Besides, anti-tumor effects have been demonstrated. However, few animal experiments and no clinical trials have been conducted thus far. This review summarizes the current knowledge on the pharmacology, ethno pharmacology, effectivity against infectious pathogens and tumors cells and the mechanism of action of the Dermaseptins. Future research should focus on further clarification of the mechanisms of action, the effectivity of Dermaseptins against several cancer cell lines and their applicability in humans.

## Introduction

Dermaseptins (DRSs) are a family of peptides that are part of the skin secretions of several *Hylid* frogs, particularly from the *Agalychnis* and *Phyllomedusa* family (Nicolas and Amiche, 2006; Amiche et al, 2008). In 1991, the first DRS was identified and characterized as a peptide rich in basic amino acids with a high propensity to adopt an α-helical structure in a hydrophobic medium (Mor et al, 1991). The DRS-S1 was purified by reverse phase high-pressure liquid chromatography (RP-HPLC) from a skin extract of the *Phyllomedusa sauvagii* and sequenced by Edman’s method. By now, there are more than a hundred DRS-like peptides classified in the large family of DRSs that share a strong identity in the cDNA sequences encoding their biosynthetic precursors (Nicolas and El Amri, 2009).

DRSs are often classified as antimicrobial peptides (AMPs), since they show effectivity *in vitro* against some gram positive and gram negative bacteria, parasites, yeasts, protozoa, viruses and display immune modulatory effects (Mor et al., 1994a; Mor et al., 1994b; Strahilevitz et al., 1994; Charpentier et al., 1998; Brand et al., 2002; Navon-Venezia et al., 2002; Brand et al., 2006; Conceicao et al., 2006; Conlon et al., 2007; Leite et al., 2008; Galanth et al., 2009; Nicolas and El Amri, 2009; Jiang et al., 2014; Zairi et al., 2014; Huang et al., 2017). Beside these antimicrobial properties, DRSs show activity against several human cancer types (Charpentier et al., 1998; Conlon et al., 2007; Nicolas and El Amri, 2009; Galanth et al., 2009; Van Zoggel et al., 2012; Shi et al., 2016; Huang et al., 2017; Dos Santos et al., 2017; Zhu et al., 2018) and can therefore be classified as an anticancer peptide as well.

In view of the current increasing bacterial resistance to conventional antibiotics (Laxminarayan et al., 2013) the demand for novel antibacterial pharmaceuticals is high. Likewise, there is a need for novel anti-tumor treatments as cancer is rapidly becoming the leading cause of death in the Western world and conventional therapeutics are both cytotoxic and prone to therapy resistance due to microevolution of the tumor tissue (Corrie, 2008; Colak and Medema, 2014).

The aim of this review is to summarize the current knowledge on DRSs, to elaborate on the ethnopharmacology, the potential therapeutic values with respect to their anti-microbial and anti-tumor potency, and to suggest future directions for research. We thereby restricted ourselves to DRSs (sensu stricto) according to the nomenclature proposed by Amiche et al. (2008).

## Ethnopharmacology in Peptide Discovery

Many plants and animal products have yet found their way from traditional use to Western medicine leading to the discovery of for example morphine, codeine, quinine, aspirin, curare, pilocarpine and ACE-inhibitors (Bisset, 1991; Alves and Alves, 2011; Dias et al., 2012). Likewise, DRS-B’s from the *Phyllomedusa bicolor* secretions are yet known to be applied for human use in traditional medicine. This *Phyllomedusa bicolor* is an Amazonian amphibian found in the forests of Brazil, the Guianas, Venezuela, Colombia, Peru, and Bolivia. The secretions of this frog are referred to by ‘Kambo’,’Kampu, or ‘Sapo’, and are used by natives as a medicine and part of a cleansing ritual. The use of Kambo by South American Indians was first described by Constantin Tastevin in 1925 in the Kachinaua, Kurina, and the Kanamari tribes (De Lima and Labate, 2008). Later, the traditional use of kambo was also documented in the Katukina tribe, the Mayoruna tribe, and the Matse tribe (De Lima and Labate, 2008; Gorman, 2015). Other DRS secreting frogs have not been documented to be useful in rituals.

The Kambo-ritual is characterized by an immediate and short-lived physical response followed by a longer lasting mental and minor physical effects. The Indians ‘harvest’ the frog by collecting the secretion from a live frog and transfer its secretions to a bamboo stick. The medicine may then be used to cure or prevent illness, to expel ‘panema’ (bad spirit), or even to induce an abortion (Gorman, 2015). When unlucky in hunting Kambo reportedly increases stamina, and sharpens senses during long hunts. Application involves burning dots on the skin, usually on arms and/or legs, and sticking a small dose (10 mg) ‘dot’ on the open wound. The symptoms are severe and immediate; violent nausea, vomiting, diarrhea, edema of the face and headaches. Symptoms last until the secretion is removed from the wound usually after 15–20 minutes.

Besides its traditional use in the Amazon, Kambo has found its way into the Western alternative healing scene as well. While the reports on the beneficial effects of this ritual are numerous and range from relieving symptoms of pain syndromes, autoimmune diseases, skin disease, and cancer to substance abuse and depression (Hesselink, 2018), so are the accounts of adverse effects of participating in a rite, with or without experienced guidance. These include a transient syndrome of inappropriate antidiuretic hormone secretion (Leban et al., 2016), presumed drug induced liver injury in a chronic alcoholic (Pogorzelska and Lapinski, 2017), sudden death upon chronic kambo use in which autopsy revealed underlying heart disease possibly related to reduced myocardial perfusion (Aquila et al., 2018) and delayed kambo related symptoms in a 24-year-old woman 22 hours after the ritual (Li et al., 2018).

The short-lasting effects of kambo are related to a diversity of biologically active peptides besides DRS including Phyllokinin (a bradykinin), Phyllomedusa (a tachykinin), Sauvagine (vasodilator), Caerulin (a CCK like peptide) and Deltorphin/Demorphin (opioid receptor agonists). Studies on these peptides have contributed greatly to our knowledge concerning the µ/δ-opioid and serotonin receptor (Erspamer et al., 1993), but are beyond the scope of this review. Many of the immediate effects of Kambo can be explained by peptides in the frog-secretion. Vasoactive properties of some of these peptides for example might contribute to the rapid absorption of the secretion into the fresh burn. Amongst these peptides, DRS is the primary candidate for the anti-microbial effects reported upon the use of Kambo.

## Structural and Pharmacological Analysis of the DRS Family

**Figure 1:**
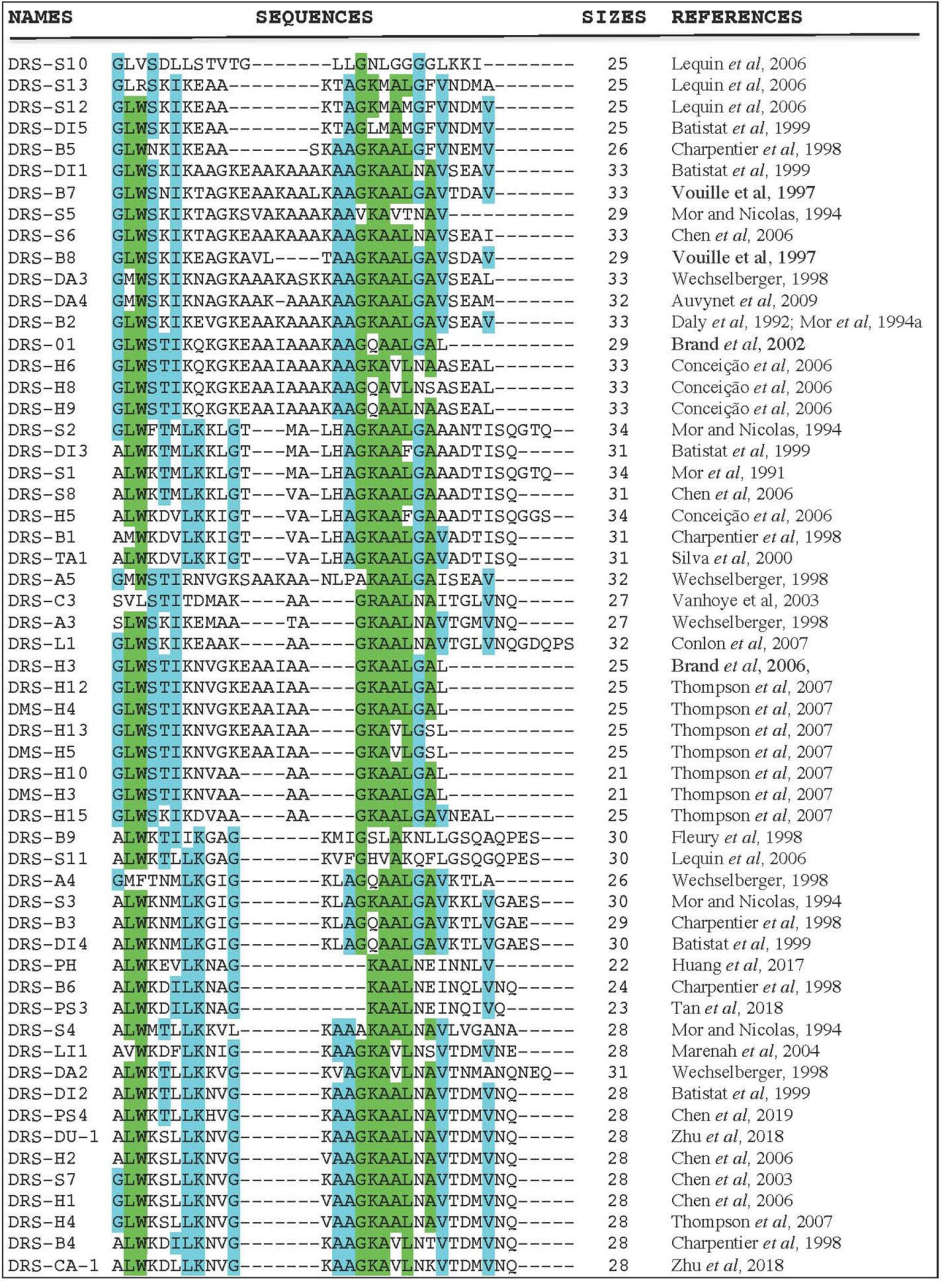
Amino acid sequence alignment of 57 DRS peptides (http://aps.unmc.edu/AP/main.php) using CLUSTAL O (1.2.4) multiple sequence alignment (www.ebi.ac.uk/Tools/msa/clustalo/). The letters represent an amino acid according to conventional nomenclature. The dashes are introduced to optimize amino-acids alignments. Blue colors indicate a well-preserved amino acid (>55% of identity), green colors indicate higher rates of preservation within this DRS group (>80% of identity).

Even though each DRS has a unique amino acid sequence and selectivity patterns towards microorganisms and tumors, there are also a lot of pharmacological similarities. **Figure 1** shows the amino acid sequence alignment of 57 DRSs sequences extracted from the antimicrobial peptide databases (http://aps.unmc.edu/AP/main.php). There is amino-acid sequence similarity within DRS from the same frog (DRS-B1–B6; 33–62%) (Charpentier et al., 1998), but also between DRS from different species (DRS-B1 and DRS-S1; 81%, DRS-B2, and DRS-D 84.8%) (Mor et al., 1994b; Auvynet et al., 2008). They are mostly rather short peptides (21–34 residues) with a highly-preserved tryptophan residue on the 3rd position from the N-terminus (Nicolas and El Amri, 2009) except for DRS-S10, DRS-S13, DRS-C3, and DRS-A4. DRSs can be fitted into an amphipathic α-helix with their hydrophobic residues on one face and the polar cationic residues in cluster on the opposite face. They usually do this is an anionic environment, or under the influence of certain phospholipids (Hoskin and Ramamoorthy, 2008). These charged clusters tend to differ among DRS, for example; DRS-B1 has a narrow polar face of a mean radial angle of 115°, DRS-B2 has a polar face covering almost half of the helix; 175° and DRS-S1 a polar face of 145° (Mor et al., 1994a). Furthermore, they show great variation in net charge and density of charge. DRS-S9 seems to be an exception as it has a highly hydrophobic core flanked by cationic residues (Lequin et al., 2006; Caillon et al., 2013). Thus far, several DRS have been *in vitro* tested for their activity against various microorganisms (Lorin et al., 2005; Savoia et al., 2010; de Moraes et al., 2011; van Zoggel et al., 2012a; Zairi et al., 2014), DRS-B2 has been tested in mice and rats in vivo for tumor and antimicrobial activity (Navon-Venezia et al, 2002; Huang et al., 2017). Though reportedly administered to humans in non-experimental settings (DRS-B’s), robust data on pharmacokinetics, efficacy and safety in humans are currently lacking.

## Research in Infectious Diseases

### Antibacterial Activity of Peptides From the DRS Family

In 1991, Mor was the first to publish on the anti-microbial properties of DRS (Mor et al., 1991). DRS-B1 and S1 show *in vitro* activity against gram positive and negative bacteria with various specificities (Strahilevitz et al., 1994). Derivatives of DRS-S4, DRS-CA1, DRS-DU1 and DRS-PH show *in vitro* activity against *Staphylococcus aureus* (including the methicillin resistant strain), *Pseudomonas aeruginosa* and *E. coli*, even when they are formed in biofilm (Navon-Venezia et al., 2002; Zairi et al., 2014; Liu et al., 2017; Zhu et al., 2018). Though less cytotoxic compared to conventional antibiotics (Zairi et al., 2014), the DRS-S4 derivatives used demonstrated similar or even higher efficacy *in vitro*(Porat et al., 2006; Rotem et al., 2006; Marynka et al., 2007; Jiang et al., 2014). These results were confirmed *in vitro* and *in vivo* with mice infected with *P. aeruginosa* (Navon-Venezia et al., 2002; Marynka et al., 2007) and in various incubation media varying in temperature and acidity (Rydlo et al., 2006). **Table 1** provides an overview of the activity of a few DRSs against selected pathogens frequently related to infections and associated with anti-biotic resistance. For a complete list of DRSs and pathogens with corresponding MIC and source, we refer to **Supplementary Table 1s**.

**Table 1:** Effectivity of Dermaseptins *in vitro* against various pathogens.

	*E. coli*	*E. faecalis*	*P. aeruginosa*	*S. aureus*	*C. albicans*	*A. fumigatus*	Human erythrocytes
DRS-B1							
DRS-B2							
DRS-B3							
DRS-B4							
DRS-S1							
DRS-S2							
DRS-S3							
DRS-S4							
DRS-S4 K4-S4(1-16)							
DRS-S5							
DRS-PH							
DRS-H3							
DRS-L1							
DRS-O1							
DRS-DI06							
DRS-CA1							
DRS-DU1							
DRS-PD1							
DRS-PD2							
DRS-PS4							

This table gives an overview of the effectivity of DRS activity against a selected number of pathogens. Cells are colored dark green if: more than one study agrees on high activity (MIC <10 μM); light green if: 1 study finds high activity (MIC <10 μM); orange if studies do not agree on MIC; red if one or more studies agree on low activity (MIC > 10 μM); white indicates that there was no published data available. In the Human erythrocyte column, the opposite is instinctively true; green color indicates low, and red indicates high activity (Strahilevitz et al., 1994; Mor et al., 1994a; Mor et al., 1994b; Carpentier et al., 1998; Brand et al., 2002; Navon-Venezia et al., 2002; Conceicao et al., 2006; Brand et al., 2006; Conlon et al., 2007; Galanth et al., 2009; Zairi et al., 2014; Huang et al., 2017; Nicolas and El Amri, 2009; Leite et al., 2008; Jiang et al., 2014; De Assis et al., 2016; Shi et al., 2016; Belmadani et al., 2018; Zhu et al., 2018; Shams et al., 2019) for more information, see **Supplementary Table S1**.

### Antiviral Activity of Peptides From the DRS Family

In addition to the antibacterial efficacy, DRS-S1 (and derivatives) show activity against pathogens causing genital infections such as human papilloma virus (HPV) and herpes simplex virus (HSV) (Savoia et al., 2010). Furthermore, modified DRS-S4 has shown antiviral activity against Herpes Simplex Virus 1 & 2, including the acyclovir resistant strain *in vitro* (Belaid et al., 2002; Bergaoui et al., 2013). For the activity peaks in case of DRS administration prior to incubation with the virus, interference early in the viral replication cycle is hypothesized (Belaid et al., 2002; Mechlia et al., 2018). DRS-S4 and S9 both demonstrate *in vitro* activity against HIV-1 virus (Lorin et al., 2005; Wang et al., 2010) by inhibition of HIV attachment to endometrial cells, uptake by the dendritic cells and subsequent transmission to T-cells (Lorin et al., 2005). Again, interference of DRS-S4 in an early phase of virus replication is suggested as less reduction of HIV is observed once the T-cells have been infected. Substitution of methionine for lysine on the 4th position DRS-S4 to reduce cytotoxicity to mammalian cells, did not affect the anti-HIV activity observed (Lorin et al., 2005). More recently, DRS-S4 has shown effectivity against Rabies virus in mice (Mechlia et al., 2018).

### Antifungal Activity of Peptides From the DRS Family

DRSs also show activity against fungi *in vitro*. So far, DRS-B1-B2, DRS-S1-S5 DRS-O1, DRS-CA1 DRS-DU1 all demonstrate cytotoxicity against *Candida albicans* (Strahilevitz et al., 1994; Mor et al., 1994a; Mor et al., 1994b; Leite et al., 2008; Nicolas and El Amri, 2009; Shi et al., 2016; Huang et al., 2017; Zhu et al., 2018). In particular, DRS-S3 shows anti-fungal activity by means of triggering apoptosis (Morton et al., 2007). In *Aspergillus fumigates*, cytotoxic activity was demonstrated for DRS-B1-B2 and DRS-S1, but not DRS-S5. Minimal inhibitory concentrations (MIC) ranging from 3.1 µM to 30 µM were observed. In addition, others (Belmadani et al., 2018) found that DRS-S1 inhibits *C. albicans* in biofilm formation when using concentrations of 100 µM. These findings are summarized in **Table 1**.

### Antiparasitic Activity of Peptides From the DRS Family

Finally, DRS-S3 and S4 derivatives appear able to target malarial parasites within a host erythrocyte without disrupting the host (Ghosh et al., 1997; Krugliak et al., 2000; Dagan et al., 2002). In addition DRS from the *Phyllomedusa oreades* (DRS-O1) has shown activity against *Schistosoma mansoni* (de Moraes et al., 2011), *Trypanosoma cruzi* (Brand et al., 2002; Leite et al., 2005) and *Leishmania amazonesis* (Brand et al., 2006).

## Research in Oncology

Parallel to the efficacy against microbials, efficacy against tumor cells has been studied as well (Hoskin and Ramamoorthy, 2008; Balandin et al., 2016). **Table 2** summarizes the data published on the impact of DRS on selected cancer cell lines.

**Table 2:** Overview of the activity DRSs against various cancer cell types.

	HEPG2	MCF-7	U251MG	H157	MDA-MB-435S	PC-3	Human erythrocytes
DRS-B2							
DRS-B3							
DRS-B4							
DRS-PH							
DRS-L1							
DRS-CA1							
DRS-DU1							
DRS-PD1							
DRS-PD2							
DRS-PS4							

This table gives an overview of the DRS that have been tested for activity against some human cancer cell lines in vitro. Cells are colored in green if: high activity (< 10 μM to reach EC50) was found, orange if: two studies did not agree on high activity, red if they agreed on low activity (> 10 μM to reach EC50), white if: no published data was available. In the Human erythrocyte column, the opposite is instinctively true; green color indicates low, and red indicates high activity (van Zoggel et al., 2012; Dos Santos et al., 2017; Mor et al., 1994b; Nicolas and El Amri, 2009; Charpentier et al., 1998; Conlon et al., 2007; Galanth et al., 2009; Shi et al., 2016; Huang et al., 2017; Zhu et al., 2018)

DRS-B2 for example, shows a dose dependent growth inhibition of prostatic adenocarcinomas (GI50 = 0.71–2.65µM) and some pancreatic cancer cell lines. Administration of 1µM DRS-B2 was enough for 50% reduction in colony formation in both prostate adenoma and mammary carcinoma cell lines (van Zoggel et al., 2012). No growth inhibition on glioblastoma and mammary carcinoma cell lines was observed (van Zoggel et al., 2012; Dos Santos et al., 2017). At a concentration of 15 µM, no activity against stromal prostate fibroblasts and skin fibroblasts was observed, indicating low cytotoxicity in surrounding tissue (van Zoggel et al., 2012; Dos Santos et al., 2017). *In vivo*, DRS did not arrest human PC3 tumor growth in mice, but inhibited growth with more than 50% vs. controls attributed to a 24% reduction of angiogenesis in tumors of treatment groups, quantified by CD34+ positive stained endothelial cells. No side-effects or differences in total blood count were observed (van Zoggel et al., 2012).

Furthermore, DRS-PH shows IC50’s of 0.69 µM, 2.01 µM, and 2.36 µM, against breast cancer adenoma (MCF-7), non-small cell lung carcinoma (NSCLC) (H157), and glioblastoma (U251MG) *in vitro* respectively (Huang et al., 2017). For DRS-PH less efficacy was observed against PC3 cell lines, compared to the activity profile of DRS-B2. DRS-PH did also show some cytotoxicity against human dermal endothelium, and mammalian red blood cells (Huang et al., 2017).

## Mechanism of Action

The mode of action by which antimicrobial peptides kill microbes is mainly known for α-helix cationic peptides which have been extensively studied (Amiche and Galanth, 2011; Melo and Castanho, 2012; Bahar and Ren, 2013). Two models explaining the interaction of α-helical cationic AMPs with membranes have been proposed: the barrel-stave model (Ehrenstein and Lecar, 1977) and the carpet or carpet model (Pouny et al, 1992), both taken over by Shai in 1999 (Shai, 1999). The cationic antimicrobial peptides, destructured in aqueous media, adopt an α-helical structure in contact with the plasma membranes of the host cell and then interact with the negative charges of the components of the membrane surface (Zasloff, 2002). After binding, the peptide will disrupt the permeability of the membrane and either cause the death of the microorganism or enter the cell compartment and interact with intracellular targets. Note that most cationic antimicrobial peptides have a direct action on the membrane of bacteria, but some such as buforine II act intracellularly (Park et al, 1998). In most of the mechanisms described, the binding of antimicrobial peptide to the membrane is followed by permeabilization of the membrane, which alone can cause cell death, or as a step in more complex processes.

In addition, very few studies on the anti-tumour action mechanism(s) of antimicrobial peptides have been conducted. The most important results show that their oncolytic mechanisms include: (i) induction of necrosis *via* cell membrane lysis, (ii) initiation of apoptosis *via* mitochondrial membrane rupture and (iii) non-membranolytic modes of action (Ellerby et al., 1999; Chen et al., 2001; Deslouches and Di, 2017).

Below, we will elaborate on how DRSs disrupt the lipid bilayer of microbes and cancer cells, and which features are possibly responsible for DRSs affinity to microbes and cancer cells. Additionally, we will discuss how DRSs modulate host immune systems and pathogen’s gene expression. Last, we summarize the evidence of DRS acting as a receptor (ant)agonist.

### Disruption of the Lipid Bilayer

One common feature DRSs often demonstrate is their disruption of the lipid bilayer of a target cell. Early evidence for this is the depolarization of bacterial membranes *in vitro* (Fleury and Longeron, 1998). To do this, DRSs likely form tetramers in their quaternary structure that form toroidal pores. Using the planar lipid bilayer technique, it was shown that the DRS peptides accumulate in a carpet like manner on the outside of a lipid bilayer until a threshold concentration is reached, causing them to form pores in which the peptides are intercalated with the phospholipid headgroups of the membrane (Duclohier, 2006). The selectivity of these pores appears to be determined by the phospholipid headgroups of the plasma membrane. The carpet of DRSs on the outside of the lipid bilayer as well as tetramer channel with intercalated phospholipid headgroups causes the membrane to lose its integrity (Yeaman and Yount, 2003). The reason why DRS favor binding to some pathogens and tumors is still under investigation and several hypotheses exist, discussed here are the possible influence of; membrane charge, membrane sulfatation and membrane fluidity.

First DRSs are generally rather cationic peptides and thus more prone to bind negatively charged membranes. Bacteria and cancer cell membranes typically have a net negative charge, the former due to negatively charged phospholipids on inner (gram negative) and single (gram positive) membranes (Shai, 2002), the latter due to expression of phosphatidylserine and negatively charged mucin proteins (Utsugi et al., 1991; Hoskin and Ramamoorthy, 2008). Interestingly, erythrocytes have net negatively charged membranes as well, but are generally less affected by DRS (Caillon et al., 2013).

Second the sulfatation of glycosaminoglycans (GAG)’s on the membrane surface seems to be of high importance for DRS-B2’s effectivity against prostate cancer cell lines (PC3) (Dos Santos et al., 2017). Low concentrations of Chondroitin Sulfate C (CS-C), which is a sulfated GAG, contribute to the α-helical shape of DRS-B2, which is its biological active form. Interestingly, sulfated GAG’s seem to be essential to the effectivity of cell penetrating peptides (CPPs) as well (Yang et al., 2014). Furthermore, when Zhu et al. (2018) investigated the activity of DRS-DP1 and 2, they found that by introducing a TAT (GRKKRRQRRR) peptide at the N-terminal, affinity to the cell membrane and interaction with GAG’s increased.

Third, cancer cells often have increased membrane fluidity and irregularities of cell surface which contributes to membrane destabilization and could affect receptor binding and other communications between cancer cell and environment (Hoskin and Ramamoorthy, 2008). This may account for DRSs being more effective against one cancer type (PC3) than another (Leuschner and Hansel, 2004; van Zoggel et al., 2012a). It has been observed that membrane environment with a strong positive curvature strain influences the DRS-B2 into a state that facilitates insertion into the membrane (Galanth et al., 2009).

### Modulation of the Host Immune System

Besides the membrane disrupting activity, DRS likely modulates the host defense system as well. By introducing DRS-S1, neutrophils of rat and humans stimulate their microbicidal activities such as their production of reactive oxygen species (Ammar et al., 1998). Moreover, DRS-S9 is chemotactic for human leukocytes (Auvynet et al., 2008). On the other hand, DRS-S4 has been observed to bind Lipopolysaccharides (LPS), which would rather suppress activation of macrophages and decrease the production of inflammatory cytokines (Navon-Venezia et al., 2002). Furthermore, DRS also shows angiostatic activities, which may influence tumor growth (van Zoggel et al., 2010; van Zoggel et al., 2012).

### Modulation of the Pathogen’s Gene Expression

We previously mentioned that DRSs can permeate membranes of different types of cells and cell nuclei, however it is also possible that DRS changes gene expression in a pathogen cell as well. DRS-S1 Modulates the expression of *C. albicans* genes, such as the Hyphal wall protein 1 (HWP1) gene (Belmadani et al., 2018). *In vitro*, its expression was downregulated which likely accounts for the modified cell morphology that was found. Furthermore, changes in aspartic protease genes were found.

### Interaction With Cell Membrane Receptors

Lastly, it seems unlikely that there is a partner protein on the surface or cytoplasm of the tumor cells that can account for the effects of DRS. This is illustrated by the example in which DRS-B2’s activity on PC3 cell lines is almost identical when composed of only amino acids in D configuration, (Dos Santos et al., 2017). Since receptors are usually stereo-selective, it is unlikely DRS-B2 works through a receptor. However, there is evidence that some DRS can inhibit adenosine triphosphate (ATP) production through receptor binding (Laughlin and Ahmad, 2010).

## Future Perspectives

As DRSs act against a wide variety of pathogens and tumor cell lines, it is tempting to speculate on their therapeutic potential. Nevertheless, current knowledge on DRS is fragmented with respect to mechanism of action and diverse with respect to pathogens and cancer cell lines studied. Consecutively, we will discuss remaining questions on the mechanism of action, potential clinical applications and safety.

### Future Research on Available DRSs

For research on DRSs to move forward on the already investigated DRSs described in this paper, it is essential to understand the mechanism of action of a specific DRS against a pathogen to fine-tune this DRS into a molecule that can target a specific clinical problem. These potential clinical therapies include treatment of infections caused by specific micro-organisms, the treatment of infections caused by multiple micro-organisms such as skin infections, and as a component of cancer treatment. Furthermore, we discuss the current use of DRS in biotechnology.

It seems likely that the mode of action of DRSs relies, similarly to many AMPs, in part on membrane disruption of target cells. Important factors in this include the cationic nature of the peptide, and its ability to retain an α-helical shape. The presence of certain phospholipids, GAGs, and proteins contribute to this. However, the means to identify more of the cell membrane factors, including the shape and fluidity of the cell membrane are not readily available, and would require innovative tools and techniques at the interface of chemistry biology and biophysics.

DRSs can be tested for specific clinical problems *in vivo*. For example, some DRSs act against specific, anti-biotic resistant pathogens. From **Table 1** we can infer that DRS-B4, DRS-O1, DRS-DI06, DRS-CA1, DRS-DU1, DRS-PD2 and DRS-PS4 are all promising candidates for specifically targeting *P. aeruginosa*, a pan-resistant bacterium notorious for causing severe infections in hospital settings. Another example is DRS-O1 which has thus far been the only DRS to show activity against the *S. mansoni*, a neglected tropical disease in need of treatment possibilities.

On the other hand, there are some DRSs that can act against a range of pathogens. This makes some DRSs ideal for clinical applications in which several pathogens need to be targeted. Skin infections, for example in diabetic foot ulcers or catheter infections are caused by organisms such as gram-positive bacteria as well as some fungi (Schittek et al., 2008). From **Table 1** we can conclude that DRS-S3, DRS-S5, DRS-CA1, DRS-DU1, DRS-PS4, DRS-PD2 and DRS-O1 all show activity against the *S. aureus* (some including the methicillin resistant variant), as well as the *C. albicans* fungus, without damaging erythrocytes. Gomes and colleagues (2015) have already positively assessed DRS’s incorporation in cotton gauzes and show potential in fast-release medical applications. Therefore they are potential starting points for therapeutic applications in skin infections.

Some even speculate that there is potential for DRS as a contraceptive, as DRS-S4 is spermicidal (Zairi et al., 2005). DRS-S4 shows activity against HIV, and several other genital pathogens (Lorin et al., 2005; Savoia et al., 2010). However, it appears the recent literature prefers antimicrobials that are within (or close to) the human genome for the use as contraceptives (Tanphaichitr et al., 2016). Moreover, the clinical relevance of such a contraceptive as well as the high degree of effectivity that would be demanded are questionable.

There is the potential of DRS as an aiding therapeutic in cancer treatment by working in tandem with conventional chemotherapies. This idea finds its roots in a hypothesis regarding the evolutionary origins of DRSs in frogs. The idea is that DRSs function as an accessory protein that lyses cells and penetrates tissue to allow effectiveness of other neuromodulators and enzymes in frog secretions (Konig et al., 2015). This contrasts with the view that DRSs are part of an innate immune system. Indeed, there is striking similarity in the amino acid sequences of preproDRS and precursors of demorphin and deltorphin (Amiche et al., 1994) (which are powerful opioid agonists), suggesting these peptides work together to achieve a common goal. This idea might extend to clinical practice as well. Potentially DRS could be part of a drug delivery system and play similar role to other cell penetrating peptides in cancer research (Bolhassani, 2011; Bolhassani et al., 2011; Yang et al., 2014). DRS would penetrate and disintegrate cell membranes of specific cancer cells and (attached) conventional cytostatic agents may then – more effectively – affect the tumor tissue. Some support for this idea comes from a study using Cecropin A and conventional chemotherapy on leukemic patients, Cecropin A increased the effect of the conventional therapy (Hui et al., 2002). Possibly the same idea can be used when targeting microbial pathogens (Balaban et al., 2004). Potentially, red blood cells or chitosan nanoparticles could function as a potential carrier for DRS (Feder et al., 2001; Medeiros et al., 2014).

Meanwhile the field of applied biotechnology has moved forward on these developments and researchers have incorporated DRS in the genetic material of potato and citrus plants. The plants can express these peptides and protect the crops from disease (Rivero et al., 2012; Furman et al., 2013). More recently, DRS-B1 was modified (N-terminally modified and recombined respectively) to protect poplar plants and tobacco plants from infections by inserting the DRS protein in the genome of the host (Yevtushenko and Misra, 2019; Shams et al., 2019). On the one hand pesticides can now be avoided on these crops, on the other hand studies already report resistance of bacteria to anti-microbial peptides by producing positively charged molecules on the membrane and pumping AMP’s out of the cells (Joo, Fu & Otto, 2016; Andersson Hughes & Kubicek Sutherland, 2016). Genetically altering plants on a large scale could potentially endanger DRS’s use as an antibiotic in humans.

Lastly, the safety of DRS in humans remains to be investigated. Even though several DRS have been administered to mice and rats, to our knowledge, no phase-1 clinical trials have been conducted on the safety of any DRS in humans thus far. Encouragingly, many of the documented short-term adverse effects of the Kambo ritual such as nausea and tachycardia can be ascribed to other molecules in the cocktail (Erspamer et al., 1993), nevertheless the safety of the DRS remain to be shown. Researchers in many fields could benefit from the knowledge of a safe DRS peptide.

### Future Research on Novel DRSs

The investigation of other Anuran species is likely to yield anti-microbial or DRS like peptides that can contribute to illuminating the mechanism of action of these peptides and provide starting points for therapeutical treatments. According to some estimations, skin compounds have been detected and isolated from 400 anuran species, which means that more than 90% of all documented frog species still await screening (Konig et al., 2015). The isolated peptides from different species thus far are very often unique and sometimes useful which makes it very likely that new, useful and novel biomolecules await discovery.

To ensure these new molecules contribute to scientific literature in a less fragmented way, it is important to identify the questions that remain on the mechanism of action of DRS. Whenever a new DRS or a modification is tested, ideally these mechanisms should be evaluated: 1) disruption of plasma/mitochondrial membranes; 2) necrosis; 3) apoptosis; 4) mechanisms of mediated immunity; 5) membrane receptor involvement; 6) inhibition of DNA synthesis; 7) anti-angiogenic effects (Gaspar et al., 2013). Additionally, their activity against human erythrocytes and epithelial cells should be evaluated to assess clinical relevance and help understand how DRS recognize target cells.

DRS are a complex family of bioactive peptides. Accumulating evidence suggests their efficacy in a wide variety of medical applications. Despite the still puzzling mechanisms of action, DRSs are extremely suitable for specific medical problems.

## Author Contributions

EB is first author and writer of this manuscript. DD supervised the initial draft of this manuscript, helped identify the aims and research question and came up with the initial structure. MA critically reviewed the drafts several times, elaborated on the mechanisms of action, revised structure and added figures.

## Conflict of Interest

The authors declare that the research was conducted in the absence of any commercial or financial relationships that could be construed as a potential conflict of interest.
