# Supplementary material for: Dermaseptins, Multifunctional Antimicrobial Peptides: A Review of Their Pharmacology, Effectivity, Mechanism of Action, and Possible Future Directions
Source: Front Pharmacol. 2019 Nov 26;10:1421. doi: 10.3389/fphar.2019.01421 (PMC6901996; doi:10.3389/fphar.2019.01421)
Supplement: Supplementary file 1 [file Table_1.docx]

Supplementary table 1. *In vitro* actvity of dermaseptin against various pathogens

| DRS | Pathogen or cell line | Paper | MIC/unless otherwise specified | Remarks |
| --- | --- | --- | --- | --- |
| DRS B1 | *E. coli* | Strahilevitz et al. 1994 | 4.70 μM |  |
| DRS B1 | *E. coli* | Nicolas & El Amri, 2009 | 1.5 μM |  |
| DRS B1 | *E. faecalis* | Nicolas & El Amri, 2009 | 50 μM |  |
| DRS B1 | *P. aeruginosa* | Shams et al., 2019 | 34 μM |  |
| DRS B1 | *P. aeruginosa* | Nicolas & El Amri, 2009 | 6.2 μM |  |
| DRS B1 | *S.aureus* | Strahilevitz et al. 1994 | 12.5 μM |  |
| DRS B1 | *S.aureus* | Nicolas & El Amri, 2009 | 25 μM |  |
| DRS B1 | *C. albicans* | Strahilevitz et al. 1994 | 9.6 μM |  |
| DRS B1 | *C. albicans* | Nicolas & El Amri, 2009 | 10 μM |  |
| DRS B1 | *A. fumigatus* | Nicolas & El Amri, 2009 | 3.1 μM |  |
| DRS B1 | Human erythrocyte | Strahilevitz et al. 1994 | 40 μg/mL | 100% hemolysis: 1 hr |
| DRS B1 | Human erythrocyte | Nicolas & El Amri, 2009 | >250 μM | 100% hemolysis: 1 hr |
| DRS B2 | *E. coli* | Mor, Amiche & Nicolas1994 | 5 μM |  |
| DRS B2 | *E. coli* | Galanth et al., 2009 | 0.8 μM |  |
| DRS B2 | *E. coli* | Nicolas & El Amri, 2009 | 1.5 μM |  |
| DRS B2 | *E. faecalis* | Nicolas & El Amri, 2009 | 50 μM |  |
| DRS B2 | *P. aeruginosa* | Galanth et al., 2009 | 3.1 μM |  |
| DRS B2 | *P. aeruginosa* | Nicolas & El Amri, 2009 | 12.5 μM |  |
| DRS B2 | *S.aureus* | Galanth et al., 2009 | 0.7 μM |  |
| DRS B2 | *S.aureus* | Nicolas & El Amri, 2009 | 12.5 μM |  |
| DRS B2 | *C. albicans* | Mor, Amiche & Nicolas, 1994 | 5 μM |  |
| DRS B2 | *C. albicans* | Nicolas & El Amri, 2009 | 5 μM |  |
| DRS B2 | *A. fumigatus* | Mor, Amiche & Nicolas, 1994 | 20 μM |  |
| DRS B2 | *A. fumigatus* | Nicolas & El Amri, 2009 | 3.1 μM |  |
| DRS B2 | MDA-MB-435S | van Zoggel et al., 2012 | 8.06 μM | EC50 |
| DRS B2 | MDA-MB-435S | Diaconescu et al., 2018 | 15 μM | EC50 |
| DRS B2 | MDA-MB-435S | Dos Santos et al., 2017 | >10 μM | EC50 |
| DRS B2 | RAJI(3lymphoma) | van Zoggel et al., 2012 | 2.57 μM | EC50 |
| DRS B2 | PC-3 | van Zoggel et al., 2012 | 1.24 μM | EC50 |
| DRS B2 | PC-3 | van Zoggel et al., 2012 | 2 μM | EC50 |
| DRS B2 | Human erythrocyte | Mor, Amiche & Nicolas, 1994 | >75 μM | 100% hemolysis: 1 hr |
| DRS B2 | Human erythrocyte | Galanth et al., 2009 | 50 μM | 100% hemolysis: 1 hr |
| DRS B2 | Human erythrocyte | Nicolas & El Amri, 2009 | >250 μM | 100% hemolysis: 1 hr |
| DRS-B3 | *E. coli* | Charpentier et al., 1998 | 2.3-2.6 μM |  |
| DRS-B3 | *E. coli* | Nicolas & El Amri, 2009 | 1.5 μM |  |
| DRS-B3 | *E. faecalis* | Nicolas & El Amri, 2009 | 12.5 μM |  |
| DRS-B3 | *P. aeruginosa* | Charpentier et al., 1998 | 2.3-5.0 μM |  |
| DRS-B3 | *P. aeruginosa* | Nicolas & El Amri, 2009 | 3.1 μM |  |
| DRS-B3 | *S. aureus* | Charpentier et al., 1998 | 1.3 μM |  |
| DRS-B3 | *S. aureus* | Nicolas & El Amri, 2009 | 3.1 μM |  |
| DRS-B3 | MCF-7 | Charpentier et al., 1998 | 12 μM | 50% inhibition of [3H]thymidine incorporation |
| DRS-B3 | PC-3 | van Zoggel et al., 2012 | 3 μM |  |
| DRS-B3 | Human erythrocyte | Nicolas & El Amri, 2009 | >100 μM | 100% hemolysis: 1hr |
| DRS-B4 | *E. coli* | Charpentier et al., 1998 | 2.3-5.0 μM |  |
| DRS-B4 | *P. aeruginosa* | Charpentier et al., 1998 | 4.6-11.6 μM |  |
| DRS-B4 | *S. aureus* | Charpentier et al., 1998 | 3.0 μM |  |
| DRS-B4 | *MCF-7* | Charpentier et al., 1998 | 15 μM | 50% inhibition of [3H]thymidine incorporation |
| DRS-S1 | *E coli* | Mor, Amiche & Nicolas 1994 | 2 μM |  |
| DRS-S1 | *E coli* | Mor, Hani & Nicolas 1994 | 1 μM |  |
| DRS-S1 | *E coli* | Charpentier et al., 1998 | 4.6 μM |  |
| DRS-S1 | *E. faecalis* | Mor, Amiche & Nicolas 1994 | 5.0 μM |  |
| DRS-S1 | *E. faecalis* | Mor, Hani & Nicolas 1994 | 5.0 μM |  |
| DRS-S1 | *P. aeruginosa* | Mor, Amiche & Nicolas 1994 | 30 μM |  |
| DRS-S1 | *P. aeruginosa* | Charpentier et al., 1998 | 2.3-50 μM |  |
| DRS-S1 | *S. aureus* | Mor, Amiche & Nicolas 1994 | 10 μM |  |
| DRS-S1 | *S. aureus* | Mor, Hani & Nicolas 1994 | 5.0 μM |  |
| DRS-S1 | *S. aureus* | Charpentier et al., 1998 | 60 μM |  |
| DRS-S1 | *C. albicans* | Mor, Amiche & Nicolas 1994 | 20 μM |  |
| DRS-S1 | *C. albicans* | Mor, Hani & Nicolas 1994 | 10 μM |  |
| DRS-S1 | *C. albicans* | Belmadani et al., 2018 | 2.9 μM |  |
| DRS-S1 | *A. fumigatus* | Mor, Amiche & Nicolas 1994 | 30 μM |  |
| DRS-S1 | *A. fumigatus* | Mor, Hani & Nicolas 1994 | 30 μM |  |
| DRS-S1 | T. Vaginalis | Savoia et al., 2010 | 22 μM |  |
| DRS-S1 | HSV-1 | Savoia et al., 2010 | 50 μM | IC50 |
| DRS-S1 | HSV-1 | Belaid et al., 2001 | no effect |  |
| DRS-S1 | Human erythrocytes | Mor, Hani & Nicolas 1994 | >70 μM | 100% hemolysis: 1 hr |
| DRS-S1 | Human erythrocytes | Charpentier et al., 1998 | none detected | 50% inhibition of [3H]thymidine incorporation |
| DRS-S1 | Human sperm cell | Zairi et al., 2005 | 200 μg/mL | EC100 |
| DRS-S2 | *E. coli* | Mor, Hani & Nicolas1994 | 2.5 μM |  |
| DRS-S2 | *E. faecalis* | Mor, Hani & Nicolas1994 | 10 μM |  |
| DRS-S2 | *S. aureus* | Mor, Hani & Nicolas1994 | 20 μM |  |
| DRS-S2 | *C. albicans* | Mor, Hani & Nicolas1994 | 10 μM |  |
| DRS-S2 | *A. fumigatus* | Mor, Hani & Nicolas1994 | 20 μM |  |
| DRS-S2 | HSV-1 | Belaid et al., 2001 | 16 μg/ml |  |
| DRS-S2 | Human erythrocyte | Mor, Hani & Nicolas 1994 | >70 μM | 100% hemolysis: 1hr |
| DRS-S3 | *E. coli* | Mor, Hani & Nicolas 1994 | 2.5 μM |  |
| DRS-S3 | *E. faecalis* | Mor, Hani & Nicolas 1994 | 10 μM |  |
| DRS-S3 | *S. aureus* | Mor, Hani & Nicolas 1994 | 10 μM |  |
| DRS-S3 | *C. albicans* | Mor, Hani & Nicolas 1994 | 10 μM |  |
| DRS-S3 | *A. fumigatus* | Mor, Hani & Nicolas 1994 | 20 μM |  |
| DRS-S3 | HSV-1 | Belaid et al., 2001 | 16 μg/ml |  |
| DRS-S3 | Rabies virus | Mechlia et al., 2018 | 8.5 μM (IC50) |  |
| DRS-S3 | Plasmodium | Ghosh et al., 1997 | 0.8-1.5 μM (IC50) |  |
| DRS-S3 | Human erythrocyte | Mor, Hani & Nicolas 1994 | 80 μM | 100% hemolysis 1hr, confirmed in vivo |
| DRS-S4 | *E. coli* | Mor, Hani & Nicolas, 1994 | 4.0 μM |  |
| DRS-S4 | *E. faecalis* | Mor, Hani & Nicolas, 1994 | 20 μM |  |
| DRS-S4 | *P. aeruginosa* | Jiang et al., 2014 | 11.3 μM |  |
| DRS-S4 | *S. aureus* | Mor, Hani & Nicolas, 1994 | 10 μM |  |
| DRS-S4 | *C. albicans* | Mor, Hani & Nicolas, 1994 | 20 μM |  |
| DRS-S4 | *A. fumigatus* | Mor, Hani & Nicolas, 1994 | 20 μM |  |
| DRS-S4 | HSV-1 | Belaid et al., 2001 | 2 μg/ml |  |
| DRS-S4 | HSV-2 | Bergaoui et al., 2013 | 2.10-6 μM (EC50) |  |
| DRS-S4 | Rabies virus | Mechia et al., 2018 | 5.1 μM |  |
| DRS-S4 | HIV | Lorin et al., 2005 | 2.0 μM (IC50) |  |
| DRS-S4 | Plasmodium | Ghosh et al., 1997 | 0.27-2.0 μM |  |
| DRS-S4 | Human erythrocyte | Mor, Hani & Nicolas 1994 | 1 μM | 100% hemolysis 1 hr, Confirmed in vivo |
| DRS-S4 K4-S4(1-16) | *E. coli* | Navon-Venezia et al., 2002 | 2 μM |  |
| DRS-S4 K4-S4(1-16) | *E. coli* | Zairi et al., 2014 | 3.12 μg/ml |  |
| DRS-S4 K4-S4(1-16) | *P. aeruginosa* | Navon-Venezia et al., 2002 | 4 μM |  |
| DRS-S4 K4-S4(1-16) | *P. aeruginosa* | Zairi et al., 2014 | 12.5 μg/ml |  |
| DRS-S4 K4-S4(1-16) | *S. aureus* | Navon-Venezia et al., 2002 | 2 μM |  |
| DRS-S4 K4-S4(1-16) | *S. aureus* | Zairi et al., 2014 | 25 μg/ml |  |
| DRS-S5 | *E. coli* | Mor, Hani & Nicolas, 1994 | 35 μM |  |
| DRS-S5 | *E. faecalis* | Mor, Hani & Nicolas, 1994 | 40 μM |  |
| DRS-S5 | *S. aureus* | Mor, Hani & Nicolas, 1994 | 2.0 μM |  |
| DRS-S5 | *C. albicans* | Mor, Hani & Nicolas, 1994 | 10 μM |  |
| DRS-S5 | *A. fumigatus* | Mor, Hani & Nicolas, 1994 | No activity |  |
| DRS-S5 | HSV-1 | Belaid et al., 2001 | 32 μg/ml |  |
| DRS-S5 | Human erythrocyte | Mor, Hani & Nicolas, 1994 | >90 μM | 100% hemolysis: 1hr |
| DRS-PH | *E. coli* | Huang et al., 2017 | 16 μM |  |
| DRS-PH | *E. faecalis* | Huang et al., 2017 | 32 μM |  |
| DRS-PH | *P. Auruginosa* | Huang et al., 2017 | 64 μM |  |
| DRS-PH | *S. aureus* | Huang et al., 2017 | 32 μM |  |
| DRS-PH | *C. albicans* | Huang et al., 2017 | 16 μM |  |
| DRS-PH | U251 | Huang et al., 2017 | 2.36 μM | EC50 |
| DRS-PH | H157 | Huang et al., 2017 | 2.01 μM | EC50 |
| DRS-PH | MCF-7 | Huang et al., 2017 | 0.69 μM | EC50 |
| DRS-PH | MDA-MB435S | Huang et al., 2017 | 9.94 μM | EC50 |
| DRS-PH | HMEC-1 | Huang et al., 2017 | 4.85 μM | EC50 |
| DRS-PH | PC3 | Huang et al., 2017 | 11.8 μM | EC50 |
| DRS-PH | Horse erythrocyte | Huang et al., 2017 | 32 μM | 100% hemolysis: 1hr |
| DRS-H3 | *E. coli* | Conceicao et al., 2006 | 6 μM | 100% growth inhibition |
| DRS-H3 | *E. coli* | Brand et al., 2006 | 6.6 μM |  |
| DRS-H3 | *P. Auruginosa* | Conceicao et al., 2006 | 6 μM | 100% growth inhibition |
| DRS-H3 | *S. aureus* | Conceicae et al., 2006 | 6 μM | 100% growth inhibition |
| DRS-H3 | *S. aureus* | Brand et al., 2006 | 26.5 μM |  |
| DRS-H3 | Human erythrocyte | Conceicao et al., 2006 | 6 μM | 0% hemolysis: 3hr |
| DRS-L1 | *E. coli* | Conlon et al., 2007 | 8 μM |  |
| DRS-L1 | *S. aureus* | Conlon et al., 2007 | >128 μM |  |
| DRS-L1 | HEPG2 | Conlon et al., 2007 | 45 μM | EC50 |
| DRS-L1 | Human erythrocyte | Conlon et al., 2007 | 200 μM | EC50 |
| DRS-O1 | *E. coli* | Brand et al., 2002 | 6.42 μM |  |
| DRS-O1 | *P. Auruginosa* | Brand et al., 2002 | 6.42 μM |  |
| DRS-O1 | *S. aureus* | Brand et al., 2002 | 3.2-12.83 μM |  |
| DRS-O1 | *S. aureus* | Leite et al., 2008 | 5.7 μM |  |
| DRS-O1 | *C. albicans* | Leite et al., 2008 | 5.7 μM |  |
| DRS-O1 | *Trypanosoma cruzi* | Brand et al., 2002 | 3 μg/ml |  |
| DRS-O1 | *Schistoma mansoni* | de Moraes et al., 2011 | 50 μg/ml |  |
| DRS-O1 | human erythrocyte | Brand et al., 2002 | 128 μg/ml | EC50 |
| DRS-O1 | leukocytes in vivo | Leite et al., 2008 | no significant decrease |  |
| DRS-CA1 | *E. coli* | Zhu et al., 2018 | 4 μM |  |
| DRS-CA1 | *E. faecalis* | Zhu et al., 2018 | 128 μM |  |
| DRS-CA1 | *P. aeruginosa* | Zhu et al., 2018 | 8 μM |  |
| DRS-CA1 | *S. aureus* | Zhu et al., 2018 | 4 μM |  |
| DRS-CA1 | *S. Areus* (methilicin resistant) | Zhu et al., 2018 | 8 μM |  |
| DRS-CA1 | *C. albicans* | Zhu et al., 2018 | 4 μM |  |
| DRS-CA1 | U251MG | Zhu et al., 2018 | >100 μM | EC50 |
| DRS-CA1 | H157 | Zhu et al., 2018 | >100 μM | EC50 |
| DRS-CA1 | MCF-7 | Zhu et al., 2018 | >100 μM | EC50 |
| DRS-CA1 | PC-3 | Zhu et al., 2018 | >100 μM | EC50 |
| DRS-CA1 | HMEC-1 | Zhu et al., 2018 | >100 μM | EC50 |
| DRS-CA1 | Horse Erythrocyte | Zhu et al., 2018 | 114 μM | EC50 |
| DRS-DU1 | *E. coli* | Zhu et al., 2018 | 4 μM |  |
| DRS-DU1 | *E. faecalis* | Zhu et al., 2018 | 64 μM |  |
| DRS-DU1 | *P. aeruginosa* | Zhu et al., 2018 | 4 μM |  |
| DRS-DU1 | *S. aureus* | Zhu et al., 2018 | 4 μM |  |
| DRS-DU1 | *S. aureus* (Methicillin resistant) | Zhu et al., 2018 | 8 μM |  |
| DRS-DU1 | *C. albicans* | Zhu et al., 2018 | 4 μM |  |
| DRS-DU1 | U251MG | Zhu et al., 2018 | >100 μM | EC50 |
| DRS-DU1 | H157 | Zhu et al., 2018 | 8.43 μM | EC50 |
| DRS-DU1 | MCF-7 | Zhu et al., 2018 | >100 μM | EC50 |
| DRS-DU1 | PC-3 | Zhu et al., 2018 | 21.6 μM | EC50 |
| DRS-DU1 | HMEC-1 | Zhu et al., 2018 | >100 μM | EC50 |
| DRS-DU1 | Horse erythrocyte | Zhu et al., 2018 | 216 μM | EC50 |
| DRS-PD1 | *E. coli* | Shi et al., 2016 | 19.6 μM |  |
| DRS-PD1 | *P. aeruginosa* | Shi et al., 2016 | 19.6 μM |  |
| DRS-PD1 | *S. aureus* | Shi et al., 2016 | 39.2 μM |  |
| DRS-PD1 | *C. albicans* | Shi et al., 2016 | 39.2 μM |  |
| DRS-PD1 | U251MG | Shi et al., 2016 | 15.08 μM | EC50 |
| DRS-PD1 | HMEC-1 | Shi et al., 2016 | 36.35 μM | EC50 |
| DRS-PD1 | Horse erythrocyte | Shi et al., 2016 | >156.6 μM | EC50 |
| DRS-PD2 | *E. coli* | Shi et al., 2016 | 5.0 μM |  |
| DRS-PD2 | *P. aeruginosa* | Shi et al., 2016 | 2.5 μM |  |
| DRS-PD2 | *S. aureus* | Shi et al., 2016 | 5.0 μM |  |
| DRS-PD2 | *C. albicans* | Shi et al., 2016 | 10.1 μM |  |
| DRS-PD2 | U251MG | Shi et al., 2016 | 13.43 μM | EC50 |
| DRS-PD2 | H157 | Shi et al., 2016 | 6.43 μM | EC50 |
| DRS-PD2 | PC-3 | Shi et al., 2016 | 3.17 μM | EC50 |
| DRS-PD2 | HMEC-1 | Shi et al., 2016 | 27.28 μM | EC50 |
| DRS-PD2 | Horse erythrocyte | Shi et al., 2016 | >161 μM | EC50 |
| DRS-PS4 | *E. coli* | Chen et al., 2019 | 8 μM |  |
| DRS-PS4 | *E. faecalis* | Chen et al., 2019 | 32 μM |  |
| DRS-PS4 | *P. aeruginosa* | Chen et al., 2019 | 16 μM |  |
| DRS-PS4 | *S. aureus* | Chen et al., 2019 | 4 μM |  |
| DRS-PS4 | *S. aureus* (methicillin resistant) | Chen et al., 2019 | 8 μM |  |
| DRS-PS4 | *C. albicans* | Chen et al., 2019 | 4 μM |  |
| DRS-PS4 | U251MG | Chen et al., 2019 | 0.057 μM | EC50 |
| DRS-PS4 | H157 | Chen et al., 2019 | 0.19 μM | EC50 |
| DRS-PS4 | MCF-7 | Chen et al., 2019 | 0.67 μM | EC50 |
| DRS-PS4 | MDA-MB-435S | Chen et al., 2019 | 0.11 μM | EC50 |
| DRS-PS4 | PC-3 | Chen et al., 2019 | 0.44 μM | EC50 |
| DRS-PS4 | HMEC-1 | Chen et al., 2019 | 0.46 μM | EC50 |
